# Retrospective analysis of adverse drug reaction patterns from the national monitoring system in a tertiary care hospital in China (2019–2024)

**DOI:** 10.1515/med-2026-1393

**Published:** 2026-03-16

**Authors:** Xiao-Lei Yuan, Xiao-Yu Fang, Feng Li, Feng-Jun Wang, Qian-Qian Ma, Jun-Tao Chen, Ya-Nan Wang

**Affiliations:** Department of Pharmacy, Jieshou People’s Hospital, Fuyang, Anhui Province, China

**Keywords:** adverse drug reactions (ADRs), retrospective analysis, risk factors

## Abstract

**Objectives:**

To characterize the patterns, risk factors, and reporting trends of adverse drug reactions (ADRs) in a tertiary care hospital in China to inform targeted medication safety interventions.

**Methods:**

A single-center, retrospective analysis was conducted on 5,564 ADR reports extracted from the National Adverse Drug Reaction Monitoring System (2019–2024). Reports were assessed for causality using the WHO-UMC criteria. Descriptive statistics and chi-squared tests were employed to analyze demographics, reporting trends, causative drugs, administration routes, and clinical outcomes.

**Results:**

The mean patient age was 62.4 years, with males slightly predominating (52.5 %). ADR reporting increased markedly over the study period, with pharmacists becoming the primary reporters (93.1 % in 2024). Intravenous infusion was the most common route (67.3 %). Antineoplastic agents were the predominant drug class implicated (34.2 %), followed by anti-infectives (10.8 %) and cardiovascular drugs (9.4 %). Statistically significant associations were found between ADR outcomes and both the type of ADR (new/serious vs. general, p<0.001) and the administration route (intravenous vs. oral, p<0.001). No significant association was observed between patient age and ADR outcomes (p=0.244).

**Conclusions:**

Antineoplastic drugs and intravenous administration are key ADR risk factors. Enhanced monitoring of high-risk medications and optimized infusion protocols are needed. Limitations include retrospective single-center design and reporting biases.

## Introduction

Adverse drug reactions (ADRs) represent a critical concern in the context of clinical medication safety. Data from the World Health Organization indicates that ADRs account for 3.7–6.5 % of hospital admissions, with an estimated 60 % of these cases considered preventable through effective risk management strategies [1]. The increasing utilization of antitumor targeted agents, biologics, and polypharmacy regimens has escalated the complexity of ADR profiles, posing significant challenges to patient safety and healthcare systems [2], 3]. Despite established national pharmacovigilance networks, significant gaps persist in the characterization of local and temporal ADR patterns within specific healthcare settings, particularly concerning high-risk medications and administration routes [4], 5]. A detailed analysis of longitudinal, hospital-based ADR data is crucial to identify institution-specific risk factors, evaluate the effectiveness of existing monitoring systems, and guide the development of targeted safety strategies [6], 7].

In the present study, 5,564 ADR cases reported between 2019 and 2024 at a tertiary care hospital were retrospectively analyzed to delineate local epidemiological patterns, identify key drug- and administration-related risk factors, and assess the evolution of reporting practices. This analysis aims to provide evidence to support enhanced surveillance, optimize clinical management protocols, and contribute to the broader pharmacovigilance knowledge base in the region.

## Materials and methods

### Data collection

A total of 5,564 valid ADR reports submitted between January 2019 and December 2024 were obtained from the National Adverse Drug Reaction Monitoring System. Duplicate reports were identified and removed based on patient identifier, drug name, reaction description, and report date. Incomplete reports lacking essential information (e.g., patient age, suspected drug, or ADR description) were excluded. Data cleaning involved standardizing drug names and ADR terminology according to the WHO Adverse Reaction Terminology.

### Causality and severity assessment

Causality for each reported ADR was assessed by clinical pharmacists using the World Health Organization-Uppsala Monitoring Centre (WHO-UMC) criteria, categorized as certain, probable, possible, or unlikely. Only reports rated as ‘certain,’ ‘probable,’ or ‘possible’ were included. ADR severity was classified as ‘serious’ according to national guidelines if it resulted in death, was life-threatening, required hospitalization or prolongation of existing hospitalization, resulted in persistent or significant disability/incapacity, or caused a congenital anomaly/birth defect. ‘New’ ADRs referred to reactions not previously documented in the product labeling within China.

### Statistical analysis

Descriptive statistics were used to analyze demographic and clinical characteristics. Categorical variables were compared using the Chi-squared (χ^2^) test. Associations between potential risk factors and ADR outcomes were examined through these bivariate analyses. A two-tailed p-value<0.05 was considered statistically significant.

### Ethical approval

This retrospective study utilized anonymized adverse drug reaction data retrieved from the national reporting system and posed no risks to participants’ rights or health. As no direct patient intervention was involved and all data were de-identified, the requirement for informed consent was waived by the ethics committee. The study protocol was reviewed and approved by the Medical Ethics Committee of Jieshou People’s Hospital (Approval Number: [2025]0032). All procedures adhered to the principles outlined in the Declaration of Helsinki.

## Results

### Demographic and clinical characteristics of patients experiencing ADRs

The clinical characteristics of the 5,564 ADR cases are presented in Table 1. The mean patient age was 62.42 ± 16.58 years. Of the total cases, 2,920 (52.48 %) involved male patients and 2,644 (47.52 %) involved female patients. A history of prior ADRs was documented in 196 cases (3.52 %), while 196 cases (8.82 %) had no prior ADR history. In 4,877 cases (87.65 %), definitive ADR records were not available in their medical history. Specific medical histories or lifestyle risk factors were identified in 376 patients (6.78 %), while the remaining 5,188 cases (93.24 %) presented no such comorbidities or risk profiles.

**Table 1:** Demographic and clinical characteristics of patients with ADRs.

Groups		n (%)
Gender	Male	2,920 (52.48)
	Female	2,644 (47.52)
Past ADR history	Yes	196 (3.52)
	No	491 (8.82)
	Not quite clear	4,877 (87.65)
Special medical histories and lifestyle profiles	Smoking history	71 (1.28)
	Alcohol use history	61 (1.11)
	Past medical history	244 (4.39)
	Other	5,188 (93.24)

### Temporal distribution of ADRs

Analysis of ADR reports from 2019 to 2024 indicated a progressive increase in reporting frequency, with the highest number of cases recorded in 2023 (1,149 cases, 20.65 %). New or serious ADRs accounted for 48.47 % of all reports, with the largest proportion observed in 2024 (797 cases, 29.55 %). Non-serious ADRs comprised 51.53 % of cases, with peak occurrence documented in 2021 (807 cases, 28.15 %) (Table 2).

**Table 2:** Distribution of ADR from 2019 to 2024.

Year	New/Serious ADRs n (%)	Non-seriousADRs n (%)	Total n (%)
2019	151 (5.60)	369 (12.87)	520 (9.35)
2020	247 (9.16)	390 (13.60)	637 (11.45)
2021	231 (8.57)	807 (28.15)	1,038 (18.66)
2022	552 (20.47)	589 (20.54)	1,141 (20.51)
2023	719 (26.66)	430 (15.03)	1,149 (20.65)
2024	797 (29.55)	282 (9.83)	1,079 (19.39)
Total (n %)	2,697 (48.47)	2,867 (51.53)	5,564 (100.00)

### Age and sex distribution of patients experiencing ADRs

Demographic analysis of ADR cases indicated distinct age- and sex-related patterns. Most ADRs were reported among patients aged 60–79 years (2,813 cases, 50.55 %), with patients aged ≥60 years comprising 62.05 % of all cases. Male patients accounted for a higher proportion of ADR cases (2,920 cases, 52.48 %) compared with female patients (2,644 cases, 47.52 %), and this predominance was consistently observed across all age groups (Table 3).

**Table 3:** Age-sex distribution of ADR cases.

Age, years	Male n (%)	Female n (%)	Total n (%)
≤18	64 (2.19)	48 (1.82)	112 (2.01)
19–29	84 (2.88)	76 (2.87)	160 (2.88)
30–39	142 (4.86)	136 (5.14)	278 (5.00)
40–49	234 (8.01)	221 (8.36)	455 (8.18)
50–59	508 (17.40)	598 (22.62)	1,106 (19.88)
60–69	663 (22.71)	578 (21.86)	1,241 (22.30)
70–79	918 (31.44)	654 (24.74)	1,572 (28.25)
≥80	307 (10.51)	333 (12.59)	640 (11.50)
Total n (%)	2,920 (52.48)	2,644 (47.52)	5,564 (100.00)

### Distribution of ADR reporting sources

The annual volume of ADR reports exhibited an overall upward trajectory, rising from 520 cases in 2019 to 1,149 cases in 2023, representing a 121 % increase, followed by a slight decrease to 1,079 cases in 2024. A substantial shift in reporting sources was noted during this period. In 2019, the majority of reports originated from nursing staff (97.12 %), while pharmacists contributed 1.73 %. By 2024, pharmacists represented the primary reporting source (93.05 %), followed by physicians (5.56 %) and nurses (1.39 %). This transition reflects the increasing role of pharmacists in ADR surveillance (Table 4).

**Table 4:** Temporal evolution of ADR reporting sources (2019–2024).

Year	Reporting sources			Total n (%)
	Physician n (%)	Pharmacist n (%)	Nurse n (%)	
2019	6 (1.15)	9 (1.73)	505 (97.12)	520 (9.35)
2020	90 (14.13)	198 (15.38)	349 (54.79)	637 (11.45)
2021	95 (9.15)	683 (65.80)	260 (20.05)	1,038 (18.66)
2022	84 (7.36)	1,035 (90.71)	22 (1.93)	1,141 (20.51)
2023	96 (8.36)	1,034 (89.99)	19 (1.65)	1,149 (20.65)
2024	60 (5.56)	1,004 (93.05)	15 (1.39)	1,079 (19.39)
Total n (%)	431 (7.75)	3,963 (71.22)	1,170 (21.03)	5,564 (100.00)

### Distribution of the top 10 administration routes associated with ADRs

Analysis of the main administration routes associated with ADRs indicated that intravenous infusion accounted for the highest proportion of ADRs (3,719 cases, 67.34 %), followed by oral administration (1,498 cases, 27.12 %). The incidence of ADRs associated with intravenous infusion was markedly higher compared with oral administration (Table 5).

**Table 5:** Top 10 administration routes associated with ADRs.

Route of administration	Number of cases n (%)
Intravenous infusion	3,719 (67.34)
Oral administration	1,498 (27.12)
Subcutaneous injection	65 (1.18)
Intra-pump infusion	60 (1.09)
Intravenous injection	59 (1.07)
Intramuscular injection	48 (0.87)
Inhalation	36 (0.65)
Topical application administration	25 (0.45)
Intra-arterial administration	13 (0.24)
Hepatic arterial infusion	9 (0.16)
Total	5,523 (100.00)

### Distribution of the top 10 dosage forms associated with ADRs

**Figure 1:**
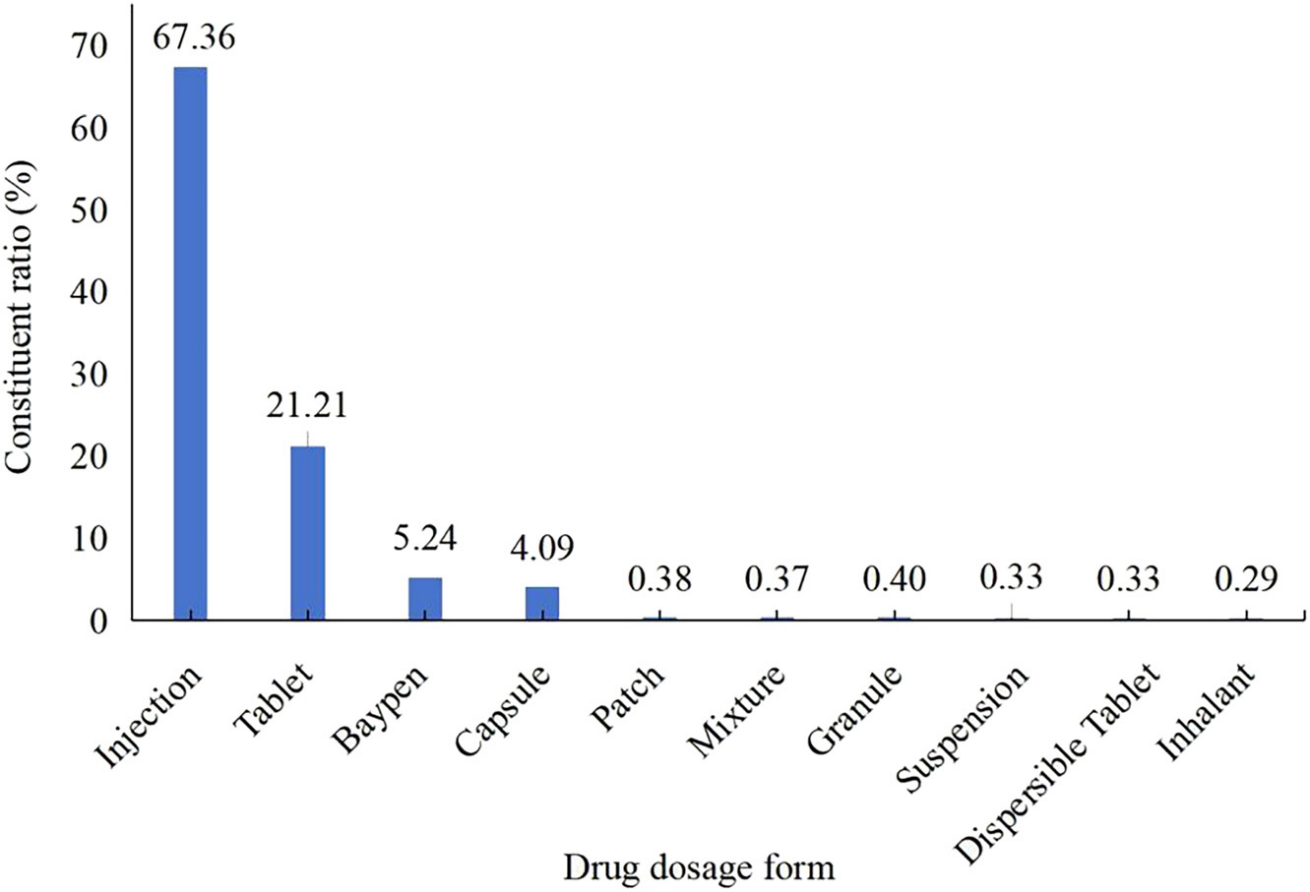
Distribution of top 10 dosage forms associated with ADRs.

As illustrated in Figure 1, injectable formulations were most frequently implicated in ADRs (67.36 %) among the top 10 implicated dosage forms, significantly exceeding all other categories. This category was followed by tablets (21.21 %), lyophilized powders for injection (5.24 %), capsules (4.09 %), and transdermal patches (0.38 %). The remaining dosage forms accounted for progressively smaller proportions of reported ADRs, reflecting a descending pattern in ADR incidence across formulation types.

### Major drug categories associated with ADRs

A total of 626 distinct drug types were implicated in ADR occurrence. Antineoplastic agents accounted for the highest proportion (1,902 cases, 34.18 %), followed by anti-infective agents (598 cases, 10.75 %) and cardiovascular agents (524 cases, 9.42 %). Within these categories, platinum-based chemotherapeutics, cephalosporins, and thrombolytic agents were the most commonly reported subtypes associated with ADRs (Table 6).

**Table 6:** Major drug categories associated with ADRs.

Drug category	Number of cases n (%)	Major agents n (%)
Antineoplastic agents	1,902 (34.18)	Carboplatin for Injection (185, 3.3 %), Gemcitabine Hydrochloride for Injection (132, 2.3 %), Oxaliplatin Injection (130, 2.3 %), Sintilimab Injection (115, 2.0 %), Capecitabine Tablets (114, 2.0 %), Cisplatin for Injection (112, 2.0 %), Paclitaxel Injection (108, 1.9 %), Albumin-Bound Paclitaxel for Injection (98, 1.7 %), et al.
Anti-infective agents	598 (10.75)	Cefoperazone-Sulbactam for Injection (100, 1.7 %), Cefuroxime Sodium for Injection (81, 1.4 %), Ceftazidime for Injection (64, 1.1 %), Piperacillin-Tazobactam for Injection (44, 0.7 %), Ciprofloxacin Lactate and Sodium Chloride Injection (40, 0.7 %), Cefoxitin Sodium for Injection (35, 0.6 %), et al.
Cardiovascular agents	524 (9.42)	Aspirin Enteric-Coated Tablets (50, 0.8 %), Rivaroxaban Tablets (32, 0.5 %), Furosemide Tablets (28, 0.5 %), Warfarin Sodium Tablets (27, 0.4 %), Rosuvastatin Calcium Tablets (26, 0.4 %), et al.
Neurological agents	487 (8.75)	Sodium Aescinate Injection (84, 1.5 %), Citicoline Sodium Injection (62, 1.1 %), Betahistine Injection (47, 0.8 %), Ginkgo Diterpene Lactone Meglumine Injection (38, 0.6 %), Ginkgolide Injection (30, 0.5 %), et al.
Gastrointestinal agents	332 (5.97)	Itopride Hydrochloride Dispersible Tablets (49, 0.8 %), Ranitidine Hydrochloride Injection (43, 0.7 %), Esomeprazole Sodium for Injection (28, 0.5 %), Pantoprazole Sodium for Injection (20, 0.3 %), Omeprazole Sodium for Injection (18, 0.3 %), et al.
Traditional Chinese medicines	246 (4.42)	Sodium Aescinate Injection (84, 1.5 %), Ginkgo Diterpene Lactone Meglumine Injection (38, 0.6 %), Ginkgolide Injection (30, 0.5 %), et al.
Respiratory agents	191 (3.43)	Doxofylline Injection (24, 0.4 %), Diprophylline Injection (24, 0.4 %), Ambroxol Injection (23, 0.4 %), Bromhexine Hydrochloride Injection (13, 0.2 %), Compound Methoxyphenamine Capsules (14, 0.2 %), et al.
Endocrine agents	161 (2.89)	Metformin Hydrochloride Sustained-Release Tablets (14, 0.2 %), Ornithine Aspartate for Injection (9, 0.1 %), Letrozole Tablets (7, 0.1 %), Metformin Hydrochloride Tablets (7, 0.1 %), Miglitol Tablets (7, 0.1 %), et al.
Contrast media	21 (0.38)	Iodixanol Injection (16, 0.2 %), Compound Meglumine Diatrizoate Injection (3, <0.1 %), Gadopentetate Dimeglumine Injection (2, <0.1 %)
Neurological agents	1,102 (19.81)	Mannitol Injection (64, 1.1 %), Lactated Ringer’s Injection (52, 0.9 %), Lornoxicam for Injection (52, 0.9 %), Compound Amino Acid Injection (18AA) (38, 0.6 %), Compound Amino Acid Injection (18AA-II) (33, 0.5 %), et al.
Total	5,564 (100.00)	Paclitaxel Injection (108, 1.9 %), Albumin-Bound Paclitaxel for Injection (98, 1.7 %), Sodium Aescinate Injection (84, 1.5 %), Ginkgo Diterpene Lactone Meglumine Injection (38, 0.6 %), Ginkgolide Injection (30, 0.5 %), et al.

### Clinical features and frequency of ADRs

**Figure 2:**
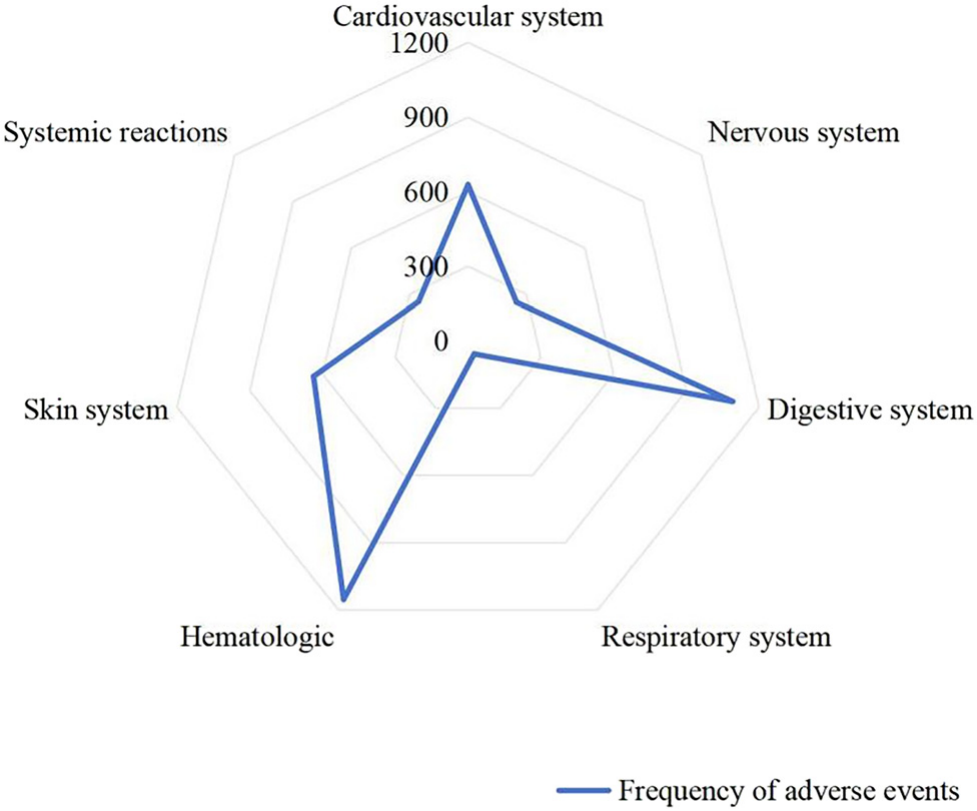
Radar map of clinical features of ADRs.

Hematologic adverse reactions constituted the most frequently reported ADR category, with bone marrow suppression and leukopenia as the predominant clinical manifestations. Gastrointestinal reactions, constituted the second most common category, typically presenting as nausea, vomiting, and diarrhea (Table7, Figure 2).

**Table 7:** Clinical features of ADRs.

Affected system	Clinical features	Frequency of ADRs
Cardiovascular	Blood pressure abnormalities, Palpitations	629
Neurological	Dizziness, Fatigue, Somnolence	249
Gastrointestinal	Nausea, Vomiting, Diarrhea	1,091
Respiratory	Dyspnea, Dry cough	57
Hematological	Bone marrow suppression, Leukopenia	1,153
Dermatological	Pruritus, Rash	637
Systemic reactions	Fever, Chills	254

### Impact of risk factors on ADRs outcomes

#### Age as a risk factor

A chi-squared (χ^2^) test was conducted to evaluate the association between age group (≤62 years vs. >62 years) and ADR outcomes. The statistical analysis indicated no significant association between age and ADR outcomes (χ^2^=4.66, p=0.244) (Table 8).

**Table 8:** Effect of age on ADRs outcomes.

Age, years	Recovery	Improvement	No improvement	Unknown	χ^2^ value	p-Value
≤62	139	1,882	82	284	4.166	0.244
>62	152	2,499	122	404		

#### ADR type as a risk factor

The relationship between ADR classification (new/serious vs. non-serious) and clinical outcomes was examined using a χ^2^ test. A statistically significant difference was identified between ADR type and outcomes (χ^2^=100.767, p<0.001), indicating a significant association between ADR classification and clinical outcomes (Table 9).

**Table 9:** Impact of ADRs type on clinical outcomes.

ADR classification	Recovery	Improvement	No improvement	Unknown	χ^2^ value	p-Value
New/Serious ADRs	197	1,976	122	402	100.767	<0.001
Non-Serious ADRs	94	2,405	82	286		

#### Route of administration

A χ^2^ test was conducted to assess the association between the route of administration (intravenous infusion vs. oral) and ADR outcomes. Statistically significant differences were identified between the intravenous infusion and oral administration groups (χ^2^=106.392, p<0.001), indicating a strong association between administration routes and ADR outcomes (Table 10).

**Table 10:** Impact of administration routes on ADR outcomes.

Route of administration	Recovery	Improvement	No improvement	Unknown	χ^2^ value	p-Value
Intravenous infusion	232	2,927	79	481	106.392	<0.001
Oral administration	52	1,149	116	181		

## Discussion

### Etiology and risk determinants of high ADR prevalence in older adult patients

Patients aged ≥60 years accounted for the majority of ADRs (62.05 %) in this cohort, a finding consistent with the high burden of age-related comorbidities (e.g., malignancies, chronic respiratory and cardiovascular diseases) in China, which necessitate the use of corresponding high-risk drug classes – antineoplastics, anti-infectives, and cardiovascular agents [8], [9], [10]. The elevated ADR susceptibility in this population is multifactorial, primarily driven by polypharmacy and multimorbidity (increasing the potential for drug-drug interactions) [11], 12], age-related pharmacokinetic decline (reduced hepatic/renal clearance leading to drug accumulation), and enhanced pharmacodynamic sensitivity to certain drug classes such as CNS agents and anticoagulants [13], 14].

Notably, multivariate analysis did not identify age as an independent predictor of serious ADR outcomes (p=0.244). This discrepancy between high incidence and non-significant outcome association may be attributed to more intensive monitoring and proactive management in geriatric care, or to limitations in outcome documentation. Thus, while age is a robust predictor of ADR occurrence, its role in determining outcome severity requires further prospective investigation.

Antineoplastic agents constituted the leading drug class (34.18 %), highlighting a critical pharmacovigilance challenge. Although novel targeted therapies often exhibit improved safety profiles, their rapid clinical adoption coupled with limited long-term real-world evidence can obscure risk recognition [15]. Mechanisms are subclass-specific: cytotoxic drugs like platinum-based agents carry narrow therapeutic indices and significant organ toxicity risks (e.g., nephrotoxicity) [16], with carboplatin being the most frequently implicated single agent in this study. Targeted therapies, such as EGFR inhibitors, are associated with mechanism-based toxicities like cutaneous reactions [17], 18].

Anti-infectives (10.75 %) and cardiovascular drugs (9.42 %) remained prominent contributors. Persistent ADR incidence from anti-infectives, despite antimicrobial stewardship efforts, suggests ongoing broad-spectrum usage patterns [19], manifesting as β-lactam hypersensitivity or fluoroquinolone-associated neurotoxicity [20]. ADRs from cardiovascular drugs, such as warfarin-related bleeding, underscore the challenges of monitoring in an aging population.

### Risk amplification effect of the intravenous administration route

Intravenous infusion accounted for 67.34 % of ADR cases in this study, with elevated risk attributable to three principal mechanisms [21], 22]. First, bypassing the first-pass effect contributes to heightened systemic drug exposure and increased susceptibility to concentration-dependent toxicities. Second, excipient-mediated reactions may occur, as immediate exposure to formulation adjuvants such as polysorbate 80 can precipitate anaphylactoid responses. Third, infusion rate–related toxicity is observed when administration kinetics are not properly controlled; for example, rapid vancomycin infusion can induce red man syndrome.

Statistically significant differences in ADR outcomes were identified between intravenous and oral administration routes (p<0.001), consistent with pharmacovigilance findings reported by DeFrancesco et al. [23]. However, the observed ADR incidence associated with oral administration (27.12 %) may represent an underestimation. This underestimation is likely influenced by delayed or latent symptom onset due to gastrointestinal absorption kinetics, as well as reporting bias, resulting from patients voluntarily discontinuing medication.

### Evolution and improvement of the ADR monitoring system

A consistent year-on-year increase in ADR reporting volume was observed from 2019 to 2024. Pharmacists became the primary reporting source during this period, with reporting shifting from nurses (21.03 %) to pharmacists (71.22 %). This transition reflects the growing maturity of the global pharmacovigilance network rather than a direct indication of deteriorating medication safety. Such trends contribute to earlier risk identification, optimization of medication regimens, and improvements in public health outcomes.

Future directions for ADR surveillance are expected to emphasize proactive early warning systems and precision prevention strategies, supported by RWE and AI–based predictive models [24], 25]. The key role of pharmacists represents a paradigm shift in hospital pharmacy services, characterized by several key improvements:

(1) Enhanced multidisciplinary collaboration: Clinical pharmacists apply therapeutic drug monitoring (TDM) and pharmacogenomic (PGx) analysis to improve ADR prediction in patients receiving complex treatment regimens.
(2) Integrated “Pharmacist–electronic heath record” systems: The use of electronic medical record triggers (e.g., abnormal liver enzyme levels [ALT>3 × ULN], Stevens–Johnson syndrome diagnostic codes) enables real-time ADR detection and targeted pharmaceutical care [26].
(3) Precision medication safety mechanisms: Individualized prevention strategies are implemented in high-risk therapeutic areas, strengthening proactive pharmacovigilance and patient safety.

### Directions for improving clinical practice

Based on the study findings, a three-pronged intervention strategy was proposed:

(1) Pre-risk control: Establishment of pharmacogenetic testing systems for antineoplastic agents to guide dose adjustments in patients with gene polymorphisms.
(2) Intra-risk intervention: Implementation of a “dual verification” protocol for intravenous administration, including assessment of infusion rate appropriateness during prescription review and vehicle compatibility testing during drug preparation.
(3) Post-risk management: Development of an ADR prediction model tailored for older adult patients, integrating parameters such as creatinine clearance, serum albumin, and polypharmacy for risk assessment. Additional measures include stepwise dosing strategies, preferential use of oral formulations when clinically appropriate, and standardized infusion protocols for essential intravenous therapies [27], [28], [29].

### Study limitations

This study has limitations. Its retrospective, single-center design may affect generalizability and introduces potential selection bias. Reliance on spontaneous reporting is susceptible to under-reporting and reporting bias, particularly for non-serious or oral drug-related ADRs. Although causality was assessed and multivariate adjustment performed, unmeasured confounders (e.g., precise dosing, detailed comorbidities) may persist. Furthermore, the study period encompasses the COVID-19 pandemic, which may have influenced drug utilization and reporting behaviors in unanalyzed ways. Future studies should strengthen risk management protocols for biologics and novel antineoplastic agents to address emerging ADR patterns [30].

## Conclusions

This retrospective study confirms that antineoplastic agents and intravenous administration are independently associated with a higher risk of serious ADRs in a tertiary care setting. The evolving role of pharmacists as key contributors to the reporting system is a positive development for institutional pharmacovigilance. These findings advocate for targeted strategies, including intensified monitoring of high-risk drug classes, optimization of intravenous administration protocols, and the continued integration of clinical pharmacists into medication safety teams to mitigate ADR risks.
